# Endogenous Ghrelin Levels and Perception of Hunger: A Systematic Review and Meta-Analysis

**DOI:** 10.1016/j.advnut.2023.07.011

**Published:** 2023-08-02

**Authors:** Kara C. Anderson, Faten Hasan, Emily E. Grammer, Sibylle Kranz

**Affiliations:** Department of Kinesiology, School of Education and Human Development, University of Virginia, Charlottesville, VA, United States

**Keywords:** hunger, appetite, ghrelin, gut hormones

## Abstract

**Background:**

Ghrelin is an orexigenic hormone primarily released by the stomach and has 2 isoforms: acylated ghrelin (AG) and de-acylated ghrelin (DAG), that appear to have different functions in humans.

**Objectives:**

To perform a systematic review and meta-analysis of the association between plasma concentrations of total ghrelin (TG), AG, and DAG and perceptions of hunger in healthy adults.

**Methods:**

The following criteria were used for inclusion: *1*) sample contained adults ≥18 y of age, *2*) body mass index [BMI kg/m^2^] was ≥18.5, *3*) ghrelin was sampled through blood, *4*) subjective hunger was measured on a validated scale, *5*) study reported a Pearson product correlation of ghrelin or had relevant figure(s) for data extraction, *6*) participants were healthy with no overt disease, *7*) protocols contained no physical activity or weight loss medication that suppressed appetite, *8*) interventions were conducted without environmental manipulations. Moderators assessed were age, BMI, percentage of body fat (%BF), macronutrient content of test meals, energy intake (kcals), sex, and ghrelin isoform (AG, DAG, or TG).

**Results:**

The analysis included 47 studies (110 trials, *n* = 1799, age: 31.4 ± 12.0 y, BMI: 26.0 ± 4.75 kg/m^2^) and measured AG (*n* = 47 trials), DAG (*n* = 12 trials), and TG (*n* = 51 trials). The overall model indicated that ghrelin concentrations and perceptions of hunger were moderately correlated (*r* = 0.43, *P <* 0.001), and ghrelin isoform significantly moderated this relationship (AG: *r* = 0.60, *P* < 0.001; TG: *r* = 0.215, *P* = 0.01; DAG: *r* = 0.53, *P* = 0.695). Other significant moderators included age (b = –0.02, *P* = 0.01), BMI (b = –0.03, *P* = 0.05), %BF (b = –0.03, *P* = 0.05), energy intake (b = 0.0003, *P* = 0.04), and percentage of carbohydrates of test meals (b = 0.008, *P* = 0.05).

**Conclusions:**

Ghrelin is associated with perceptions of hunger in humans, and this relationship is strengthened when AG is isolated; thus, AG may have a large impact on hunger signals in various populations. Future research should attempt to understand the role of DAG in hunger sensations.

**Statements of significance**

This meta-analysis is the first to assess the relationship between ghrelin isoform and perceptions of hunger. Our findings indicate the importance of distinguishing between ghrelin isoforms and highlight several gaps in the literature that should be examined in future studies.

## Introduction

Ghrelin, discovered in 1999, is the growth hormone stimulating peptide [1] predominately released from the gastric fundus and circulates in 2 isoforms: acylated ghrelin (AG) and de-acylated ghrelin (DAG), and is the only known orexigenic hormone [1]. The majority of ghrelin circulates as DAG [∼78% of total ghrelin (TG)]. The less abundant form, AG (∼22% of TG), is a post-translational modification of TG. AG is catalyzed by the enzyme ghrelin o-acyltransferase and binds to the growth hormone secretagogue receptor 1a (GHSR1a) [1] in the arcuate nucleus of the hypothalamus [1]. DAG was originally considered to be inactive because it does not bind the same receptor as AG; however, recent data suggest that there is a specific, yet unidentified DAG receptor and DAG has its own biological effects [2].

While TG concentrations fluctuate with a diurnal rhythm, secretion can be affected by meal timing and macronutrient content [3,4], and other factors such as age, sex, and BMI [3]. Because of its role in humans, TG is often termed the “hunger hormone” and has been implicated in the maintenance of energy balance, suggesting it can be a clinical target in obesity and weight management interventions. In individuals with obesity, plasma concentrations of AG appear to be reduced or increased [5,6], whereas DAG is either unchanged or elevated [6,7], highlighting the importance of measuring the isoforms of ghrelin seperately.

AG can cross the blood-brain barrier and bind to GHSR1a receptors in the area of the hypothalamus, which promotes appetite [8]. AG mediates this action by stimulating neurons that express NPY and agouti-related proteins, which are both anabolic peptides [9]. Ghrelin has also been found to suppress visceral, afferent vagal nerve activity, a conduit implicated in transmitting feeding status to the brain [9]. Observational studies indicate endogenous TG concentrations appear to rise prior to meal initiation and decrease after consumption, even in the absence of food-related cues [10], which supports its role in appetite or hunger stimulation. One of the first infusion studies in humans demonstrated that a dose comparable to normally circulating concentrations of TG (5.0 pmol/kg/min) led to a significant increase in the perception of hunger [11]. Many studies published thereafter replicated this result [12]; however, a majority of the protocols lacked a placebo group as a control, did not distinguish between isoforms, and applied supraphysiological doses of ghrelin [13]. In addition, animal models indicate that the location of ghrelin administration (e.g., peripherally or centrally) may impact the effect of ghrelin on appetite [14]. Consequently, the effects of ghrelin on hunger in response to a physiological dose or measuring endogenous ghrelin are still poorly understood. As previously mentioned, the effects of ghrelin differ by isoform; while AG has been shown to stimulate appetite in human and animal models [11,15,16], DAG has been shown to either have no effect or suppress appetite [17,18]. The distinction between the 2 isoforms has important implications for health as they have been shown to act independently, synergistically, or antagonistically with each other in a variety of tissues such as the heart, liver, pancreas, and stomach [1].

Therefore, we sought to determine if *1*) there was an association between endogenous ghrelin concentrations and perceptions of hunger in humans and *2*) if the direction and strength of this relationship differed by isoform. We also included meal content to investigate the potential effect of food intake.

Sample demographic variables were included in this analysis to explore possible moderators of this relationship. The results from this analysis will advance the scientific field, especially the potential role of ghrelin as a therapeutic target for the management of obesity and its associated comorbidities.

## Methods

This systematic review and meta-analysis were performed in accordance with PRISMA guidelines [19]. This review was not registered.

### Literature search

Electronic databases (PubMed, Medline, and Web of Science) were searched by 2 authors (KCA and FH). Articles published through November 2022 were included. The search used the following terms: (ghrelin) AND (relationship) AND (hunger). Reference lists of all relevant studies, along with reviews and book chapters, were also examined. Articles were limited to studies published in the English language.

### Article selection

For the purpose of this meta-analysis, the term “article” was used synonymously with “study” and “trial” as the unit included in the meta-analysis. Articles often contained multiple eligible trials comprised of a ghrelin and appetite measure. First, the titles and abstracts of the articles were screened for eligibility. The following criteria were determined a priori for article inclusion: *1*) sample contained adults ≥18 y of age, *2*) BMI was ≥18.5 kg/m^2^, *3*) ghrelin was sampled through blood, *4*) subjective hunger was measured on a validated scale, *5*) study reported a Pearson product correlation of ghrelin or had relevant figure(s) for data extraction, *6*) participants were healthy with no overt disease, *7*) protocols contained no physical activity or weight loss medication that suppressed appetite, and *8*) interventions were carried out without any environmental manipulations. Two authors (KCA, FH) independently completed the study selection and identified studies for inclusion. Any disparities in the initial study selection were resolved by 2 tie-breakers (EEG and SK).

### Data extraction and bias assessment

For studies that met the inclusion criteria, the following data were extracted and tabulated: *1*) author, publication year; *2*) continuous variables: sample size, age, Pearson product correlation between ghrelin and subjective hunger, BMI, %BF, and if applicable, macronutrient content of the meal [percentage of carbohydrates (%CHO), percentage of fat (%FAT), and percentage of protein (%PRO)] and EI (kcals); and *3*) categorical variables: sex, ghrelin form (AG, DAG, or TG).

If the ghrelin and hunger correlation value was not reported, the study author was contacted. When authors did not respond to follow-up, ImageJ software (NIH, Bethesda Maryland and LOCI, University of Wisconsin) [20] was utilized using data from relevant figures for manual correlation calculation using R software version 4.0.2. Data extraction was completed independently by 3 authors (KCA, FH, EEG), who demonstrated an interclass correlation coefficient of 0.99. Trials that calculated an overall appetite score but did not include an individual hunger score were excluded.

### Statistical analysis

The meta-analysis was performed using R software version 4.0.2 and the “metafor,” “metaviz,” and “ggplot2” packages, versions 3.0.2, 0.3.1, and 3.3.5, respectively [21]. The significance level for all hypothesis tests was set a priori at *P* ≤ 0.05. Descriptive data are presented as mean ± SD unless otherwise noted.

Due to the possibility of correlation variation being affected by sampling distribution skewness, correlations between ghrelin and hunger were transformed to a Fisher’s Z correlation coefficient to normalize skewness [22]. A 3-level, random effects model with restricted maximum likelihood estimation was used to account for any dependence of correlations between trials within the same study, as studies contributed multiple data points using this method [23]. This model accounts for sampling variance of the extracted effect sizes at the level of the subject (level 1), variance of the effect sizes within the same study (level 2), and variance of the extracted effect sizes between studies (level 3). The Fisher’s Z of ghrelin and hunger from each trial were inputted into the model to determine the pooled effect and associated CIs. For interpretation purposes, the pooled effect was transformed back into a correlation. The *I*^2^ statistics was used to assess the amount of heterogeneity at each level of the model, with the following interpretation: Values <25% indicate low risk of heterogeneity, 25–75% indicates moderate risk of heterogeneity, and >75% indicates considerable risk of heterogeneity [24].

Subsequent to estimating the overall effect, we examined the robustness of the pooled results via publication bias and sensitivity analysis. Sensitivity analysis was performed using Cook’s distance to determine potentially influential studies [25] (using a threshold of 4/n to determine outliers), along with excluding 1 study at a time and rerunning the pooled analysis to determine the robustness of the pooled effect. A sunset (power-enhanced) funnel plot was utilized to determine publication bias and to estimate the overall effect while accounting for the calculated power of each included trial.

Moderator analyses were used to determine which categorical variables moderated the overall pooled effect using 2 subgroups: sex and ghrelin form (TG, AG, or DAG). Fisher’s Z values from subgroup analyses were transformed back into a correlation coefficient for interpretation. Meta-regressions were also used to determine if the following continuous variables impacted the pooled correlation coefficient: Age, BMI, %BF, meal information EI, %CHO, %FAT, %PRO. The regression coefficient (ß) is reported along with 95% CI.

## Results

### Literature search

**FIGURE 1:**
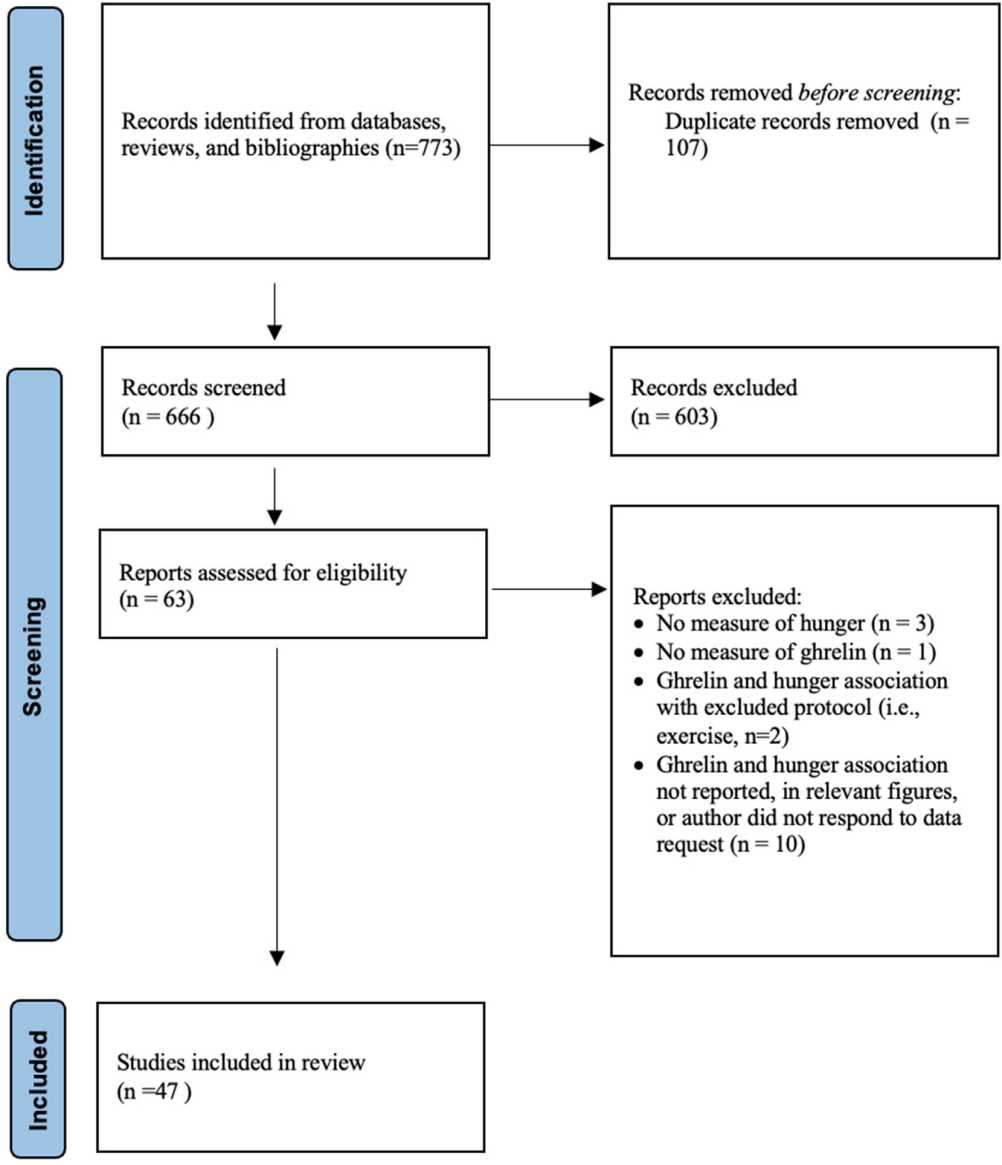
PRISMA diagram. PRISMA, preferred reporting items for systematic reviews and meta-analyses. Adapted with permission [19].

The PRISMA flow diagram outlining this process is presented in Figure 1. The initial search identified 658 articles found via database searches, with an additional 8 articles identified through reference list searches. After we screened titles and abstracts, 603 articles were excluded, which led to a full-text review of 63 eligible articles. After full-text review, 47 articles met all inclusion criteria, which contained 110 acceptable trials (and 110 correlation coefficients). The majority of trials contained males (only males *n* = 52; only females *n* = 12; both sexes *n* = 46) and measured TG (TG: *n* = 51; AG: *n* = 47; DAG: *n* = 12). The full characteristics of included trials are in Table 1 [[26], [27], [28], [29], [30], [31], [32], [33], [34], [35], [36], [37], [38], [39], [40], [41], [42], [43], [44], [45], [46], [47], [48], [49], [50], [51], [52], [53], [54], [55], [56], [57], [58], [59], [60], [61], [62], [63], [64]]. The majority of included trials used visual analog scales to measure hunger, and 2 trials used a validated visual scale that was significantly associated with visual analog scales [65,66].

**TABLE 1:** Trial characteristics

Trial	*n*	Ghrelin	Sex	Age (y)	BMI (kg/m^2^)	%BF	Meal content			
							EI (kcals)	%CHO	%FAT	%PRO
Andarini et al. [26]_a_	16	AG	M	20.6	21.25	17.0	-	-	-	-
Andarini et al. [26]_b_	16	AG	M	20.6	21.25	17.0	544.5	-	-	-
Andarini et al. [26]_c_	16	AG	M	20.6	21.25	17.0	544.5	-	-	-
Andarini et al. [26]_d_	16	AG	M	20.6	21.25	17.0	544.5	-	-	-
Andarini et al. [26]_e_	19	AG	M	21.4	21.25	33.6	-	-	-	-
Andarini et al. [26]_f_	27	AG	M	21.4	21.25	33.6	544.5	-	-	-
Andarini et al. [26]_g_	20	AG	M	21.4	21.25	33.6	544.5	-	-	-
Andarini et al. [26]_h_	24	AG	M	21.4	21.25	33.6	544.5	-	-	-
Bauer et al. [27]_a_	19	AG	M/F	80.7	26.4	-	705.0	75.0	12.0	13.0
Bauer et al. [27]_b_	19	AG	M/F	80.7	26.4	-	705.0	75.0	12.0	13.0
Bauer et al. [27]_e_	15	AG	M/F	35.4	25.3	-	705.0	75.0	12.0	13.0
Bauer et al. [27]_d_	15	AG	M/F	34.5	25.3	-	705.0	75.0	12.0	13.0
Becker et al. [28]	8	AG	M	28.0	24.0	-	817.5	69.97	20.03	10.0
Boelsma et al. [29]_a_	21	TG	M	33.0	22.4	-	675.2	60.0	30.0	10.0
Boelsma et al. [29]_b_	21	TG	M	33.0	22.4	-	675.2	35.0	30.0	35.0
Bowen et al. [30]_a_	19	TG	M	53.3	32.1	-	258.0	15.0	2.0	83.0
Bowen et al. [30]_b_	19	TG	M	53.3	32.1	-	245.0	87.0	1.0	12.0
Bowen et al. [30]_c_	19	TG	M	53.3	32.1	-	245.0	88.0	1.0	11.0
Brennan et al. [67]_a_	16	TG	M	29.0	24.0	-	1200.0	30.0	55.0	15.0
Brennan et al. [67]_b_	16	TG	M	29.0	24.0	-	1200.0	30.0	25.0	45.0
Brennan et al. [67]_c_	16	TG	M	29.0	24.0	-	1200.0	60.0	30.0	10.0
Brennan et al. [67]_d_	16	TG	M	33.0	33.0	-	1261.7	30.0	55.0	15.0
Brennan et al. [67]_e_	16	TG	M	33.0	33.0	-	1261.7	30.0	25.0	45.0
Brennan et al. [67]_f_	16	TG	M	33.0	33.0	-	1261.7	60.0	30.0	10.0
Brennan et al. [67]_g_	16	TG	M	29.0	24.0	-	1200.0	40.0	30.0	30.0
Brennan et al. [67]_h_	16	TG	M	33.0	33.0	-	1261.7	40.0	30.0	30.0
Broom et al. [65]_1_	9	AG	M	21.2	22.2	-	1041.1	38.0	52.0	10.0
Broom et al. [66]_2_	11	AG	M	21.3	23.1	-	1544.0	33.0	56.0	11.0
Cheng et al. [31]	12	TG	M	24.6	25.4	9.5	1356.3	26.0	70.0	4.0
Cummings et al. [10]	6	TG	M	21.2	21.3	12.2	1916.3	-	-	-
Deighton et al. [32]	12	AG	M	23.0	24.2	-	2473.0	64.0	15.0	21.0
Diepvens et al. [33]_a_	39	TG	M/F	42.3	27.6	32.6	244.7	64.0	15.0	21.0
Diepvens et al. [33]_b_	39	TG	M/F	42.3	27.6	32.6	244.7	64.0	15.0	21.0
Diepvens et al. [33]_c_	39	TG	M/F	42.3	27.6	32.6	244.7	64.0	15.0	21.0
Diepvens et al. [33]_d_	39	TG	M/F	42.3	27.6	32.6	244.7	64.0	15.0	21.0
Di Francesco et al. [34]_a_	8	TG	M/F	77.9	25.75	-	800.0	40.0	45.0	15.0
Di Francesco et al. [4]_b_	8	TG	M/F	29.5	24.2	-	800.0	40.0	45.0	15.0
Dorling et al. [35]_a_	12	DAG	M	20.9	23.5	15.6	1344.0	52.0	25.0	23.0
Dorling et al. [35]_b_	12	DAG	M	21.3	23.5	13.9	1228.25	52.0	25.0	23.0
Dorling et al. [35]_c_	12	AG	M	20.9	23.5	15.6	1344.0	52.0	25.0	23.0
Dorling et al. [35]_d_	12	AG	M	21.3	23.5	13.9	1228.25	52.0	25.0	23.0
Eller at al. [36]_a_	10	DAG	M	27.9	24.1	-	-	-	-	-
Eller at al. [36]_b_	10	DAG	M	27.9	24.1	-	720.0	11.0	79.0	10.0
Eller at al. [36]_c_	10	DAG	M	27.9	24.1	-	720.0	80.0	10.0	10.0
Doucet et al. [37]	25	TG	F	50.4	23.5	32.0	537.5	52.0	29.5	18.6
Erdmann et al. [38]_1a_	30	TG	M/F	37.7	35.6	-	861.0	-	-	-
Erdmann et al. [38]_1b_	30	TG	M/F	37.7	35.6	-	441.0	-	-	-
Erdmann et al. [39]_2_	14	TG	M/F	22.6	22.6	-	584.0	-	-	-
Gibbons et al. [40]_a_	16	TG	M/F	45.6	29.8	39.5	590.0	38.0	50.3	11.7
Gibbons et al. [40]_b_	16	TG	M/F	45.6	29.8	39.5	590.0	38.0	50.3	11.7
Gibbons et al. [40]_c_	16	TG	M/F	45.6	29.8	39.5	590.0	83.6	3.2	13.2
Gibbons et al. [40]_d_	16	TG	M/F	45.6	29.8	39.5	590.0	83.6	3.2	13.2
Giuntini et al. [41]_a_	52	TG	F	29.3	21.8	-	-	-	-	-
Giuntini et al. [41]_b_	52	TG	F	29.3	21.8	-	-	-	-	-
Heden et al. [68]_a_	14	AG	M/F	26.0	22.9	24.9	600.0	45.0	40.0	15.0
Heden et al. [68]_b_	14	AG	M/F	25.1	34.8	41.0	600.0	45.0	40.0	15.0
Hernandez et al. [42]_a_	22	AG	M/F	34.0	36.4		299.7	55.0	21.0	24.0
Hernandez et al. [42]_b_	22	DAG	M/F	34.0	36.4		299.7	55.0	21.0	24.0
Hernandez et al. [42]_c_	22	AG	M/F	34.0	36.4		-	-	-	-
Hernandez et al. [42]_d_	22	DAG	M/F	34.0	36.4		-	-	-	-
Kawano et al. [43]	15	AG	M	24.4	22.1	15.1	-	-	-	-
King et al. [44]_1_	9	AG	M	22.2	23.6	17.8	3854.2	69.3	8.3	23.4
King et al. [45]_2_	14	AG	M	21.9	23.4	19.2	2202.0	51	34	15
King et al. [46]_3_	10	AG	M	22.0	23.2	17.2	1579.6	61.7	18.2	19.1
King et al. [47]_4a_	12	AG	M	23.4	22.8	-	525.8	48.0	34.0	18.0
King et al. [47]_4b_	12	AG	M	23.4	22.8	-	1678.05	48.0	34.0	18.0
King et al. [47]_5_	9	AG	M	22.0	22.6	-	1152.0	48.0	33.0	19.0
Larsen-Meyer et al. [69]_a_	10	TG	F	24.6	22.1	35.7	1811.0	62.3	22.7	14.9
Larsen-Meyer et al. [69]_b_	9	TG	F	23.7	19.8	23.0	2035.0	63.6	21.4	14.1
Lejuene et al. [70]_a_	20	AG	F	21.0	21.1	24.3	-	60.0	30.0	10.0
Lejuene et al. [70]_b_	20	AG	F	21.0	21.1	24.3	-	40.0	30.0	60.0
Little et al. [48]_a_	16	TG	M/F	26.0	22.3	-	-	-	-	-
Little et al. [48]_b_	16	TG	M/F	26.0	22.3	-	750	53.8	29.5	16.7
Little et al. [48]_c_	16	TG	M/F	26.0	22.3	-	750	53.8	29.5	16.7
Little et al. [48]_d_	16	TG	M/F	26.0	22.3	-	750	53.8	29.5	16.7
Maersk et al. [49]_a_	24	AG	M/F	33.5	31.4	-	900	-	-	-
Maersk et al. [49]_b_	24	AG	M/F	33.5	31.4	-	950	-	-	-
Maersk et al. [49]_c_	24	AG	M/F	33.5	31.4	-	7.5	-	-	-
Maersk et al. [49]_d_	24	AG	M/F	33.5	31.4	-	-	-	-	-
Malkova et al. [50]	11	TG	M	23.8	23.3	13.7	932.1	-	-	-
Martins et al. [51]_1_	12	AG	M/F	33.4	32.2	-	600.0	48.0	35.0	17.0
Martins et al. [52]_2_	12	AG	M/F	25.9	22.0	-	500.0	59.4	21.4	18.9
Massolt et al. [53]_a_	12	TG	F	26.6	-	-	-	-	-	-
Massolt et al. [53]_b_	6	TG	F	26.6	-	-	-	-	-	-
Massolt et al. [53]_c_	6	TG	F	26.6	-	-	-	-	-	-
Metcalfe et al. [54]	8	AG	M	21.2	25.0	-	-	-	-	-
Sakuma et al. [55]_a_	9	DAG	M/F	26.7	22.0	18.5	299.95	100.0	0.0	0.0
Sakuma et al. [55]_b_	9	DAG	M/F	26.7	22.0	18.5	343.0	93.2	1.1	5.7
Sakuma et al. [55]_c_	9	DAG	M/F	26.7	22.0	18.5	348.9	92.0	1.3	6.7
Sakuma et al. [55]_d_	9	DAG	M/F	26.7	22.0	18.5	353.0	91.2	1.7	7.1
Sakuma et al. [55]_e_	9	DAG	M/F	26.7	22.0	18.5	362.1	89.4	2.0	8.6
Sim et al. [56]	17	AG	M	30.0	27.7	30.0	267.7	61.0	30.0	15.0
Stubbs et al. [57]_a_	15	TG	M/F	28.0	22.0	-	-	-	-	-
Stubbs et al. [57]_b_	15	TG	M/F	28.0	22.0	-	-	-	-	-
Vatansever-Ozen et al. [58]	10	AG	M	20.1	22.0	16.5	-	-	-	-
Wasse et al. [59]_1_	10	AG	M	24.0	24.8	-	1800.9	47.0	37.0	16.0
Wasse et al. [60]_2_	12	AG	M	22.7	23.4	18.6	831.9	33.0	56.0	11.0
Willis et al. [61]_a_	20	TG	M/F	26.0	24.0	-	504.0	51.4	13.5	7.6
Willis et al. [61]_b_	20	TG	M/F	26.0	24.0	-	488.0	46.0	7.4	6.8
Willis et al. [61]_c_	20	TG	M/F	26.0	24.0	-	493.0	50.85	7.4	6.85
Willis et al. [61]_d_	20	TG	M/F	26.0	24.0	-	544.0	45.6	6.4	6.4
Wuorinen et al. [62]_a_	10	TG	F	59.5	25.85		1212.0	60.0	25.0	15.0
Wuorinen et al. [62]_b_	10	TG	F	57.2	23.4		1606.0	60.0	25.0	15.0
Yau et al. [63]_a_	7	AG	M	25.0	25.5	21.0	-	-	-	-
Yau et al. [63]_b_	7	AG	M	25.0	25.5	21.0	158.4	100.0	0.0	0.0
Yau et al. [63]_c_	7	AG	M	25.0	25.5	21.0	144.0	100.0	0.0	0.0
Yau et al. [63]_d_	7	AG	M	25.0	25.5	21.0	144.0	100.0	0.0	0.0
Yau et al. [63]_e_	7	AG	M	25.0	25.5	21.0	151.2	100.0	0.0	0.0
Zhu et al. [64]_a_	31	TG	M/F	38.0	29.0	-	637.0	75.7	14	11.5
Zhu et al. [64]_b_	28	TG	M/F	37.9	28.8	-	642.0	50.3	43	10.4

Numerical subscript indicates a different study, letter subscript indicates a different trial within the same study.

AG, acylated ghrelin; DAG, de-acylated ghrelin; TG, total ghrelin; %CHO, percentage of carbohydrates; %PRO, percentage of protein.

### Pooled effect

**FIGURE 2:**
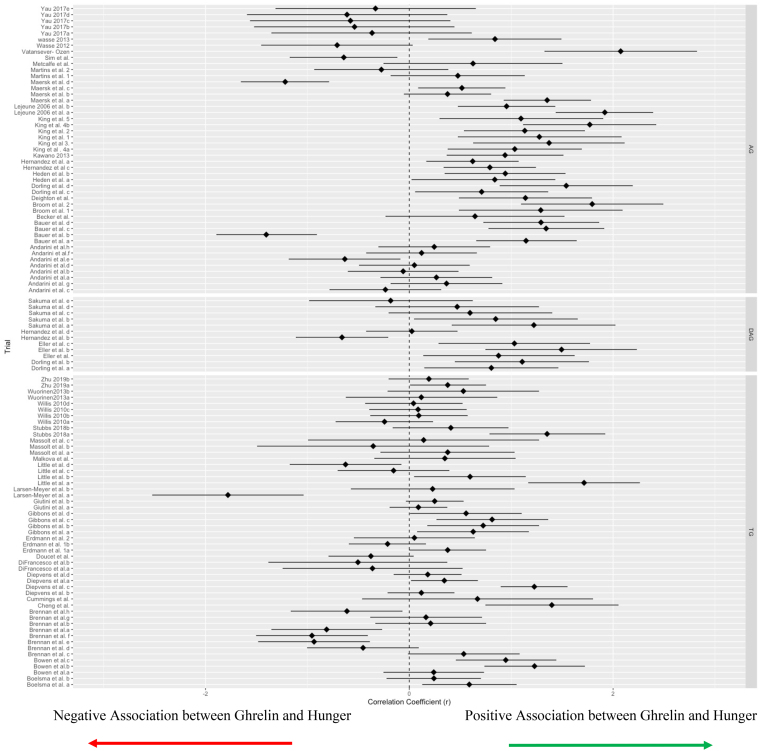
Forest plot of the overall effect by ghrelin isoform.

The overall model indicated that ghrelin concentrations and subjective hunger ratings had a positive, moderate association (Fisher’s Z = 0.47 (which transforms back to a correlation coefficient of 0.43), 95% CI: 0.28–0.57; *P* < 0.001, Figure 2). The overall model had significant heterogeneity with substantial within-study (level 2) heterogeneity (I^2^ = 56.4%, *P* < 0.0001) and moderate between-study (level 3) heterogeneity (I^2^ = 30.0%, *P* < 0.003).

### Cook’s distance and power-enhanced funnel plot

**FIGURE 3:**
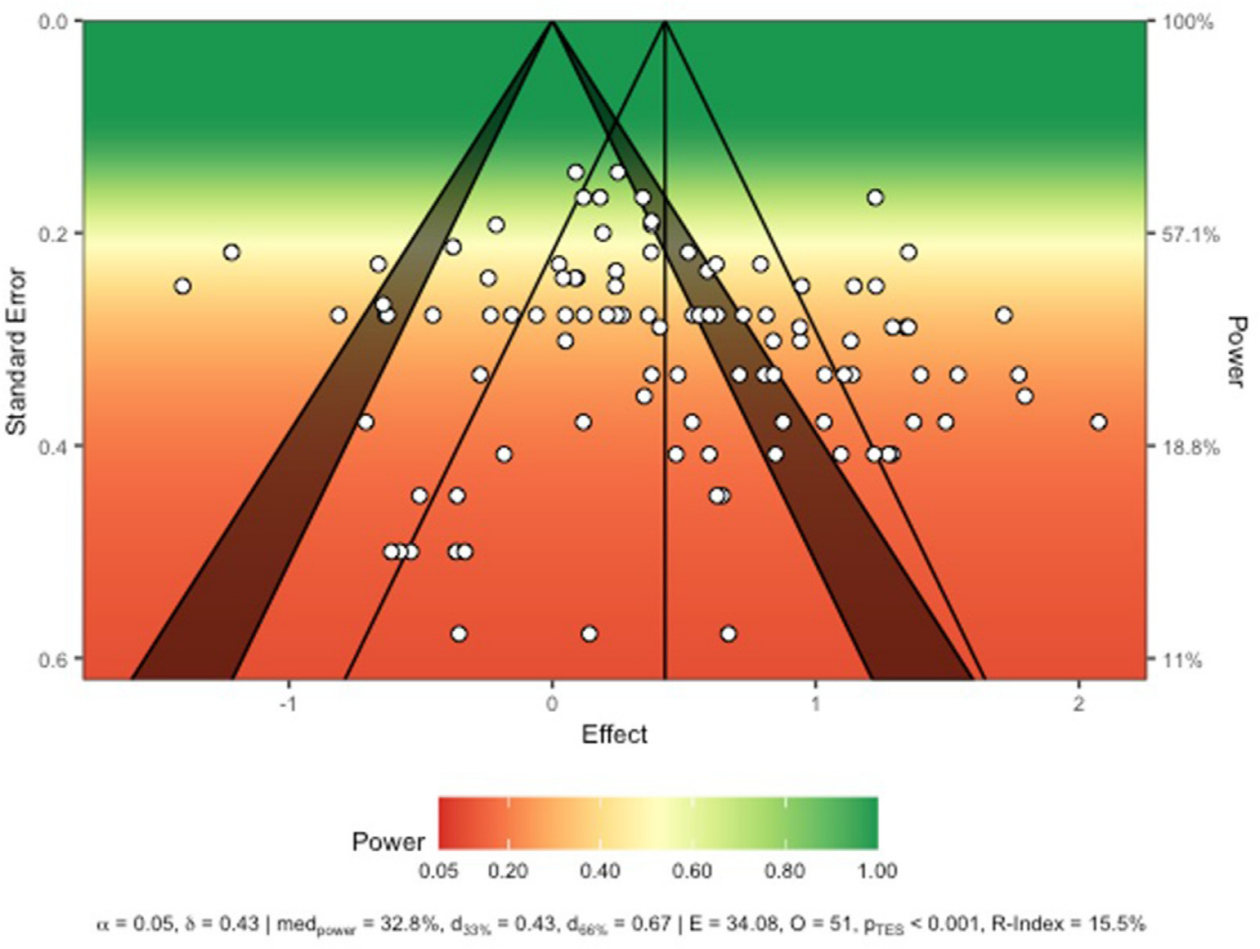
Sunset enhanced funnel plot.

Cook’s distance identified several influential studies [[67], [69], [70]] (Supplemental Figure 1). When these studies were removed from the pooled analysis, the association between ghrelin concentrations and hunger remained significant, while the relationship changed minimally *(r* = 0.44, *P* < 0.01; level 2 I^2^ = 59.5%, *P* < 0.0001; I^2^ level 3 = 25.0%, *P* < 0.0001). It is unclear why these studies had such an effect on the model beyond that most of them contained trials with negative correlations and/or strong correlations. All other studies removed during the sensitivity analysis had no substantial effect on the overall model. Visual inspection of the sunset plot (Figure 3) revealed asymmetry - a majority of included trials were underpowered (median power = 32.8%). Based on the trials’ power, Fisher’s Z was estimated at 0.43 (*r* = 0.40), and the R-Index was calculated at 15.5%, which suggests a low probability of replication. The Fisher’s Z needed for achieving 66% of median power is 0.67. Given the data, we would expect 34.08 statistically significant trials, and we observed 51, yielding a *P* value of <0.001.

### Moderator analyses

**FIGURE 4:**
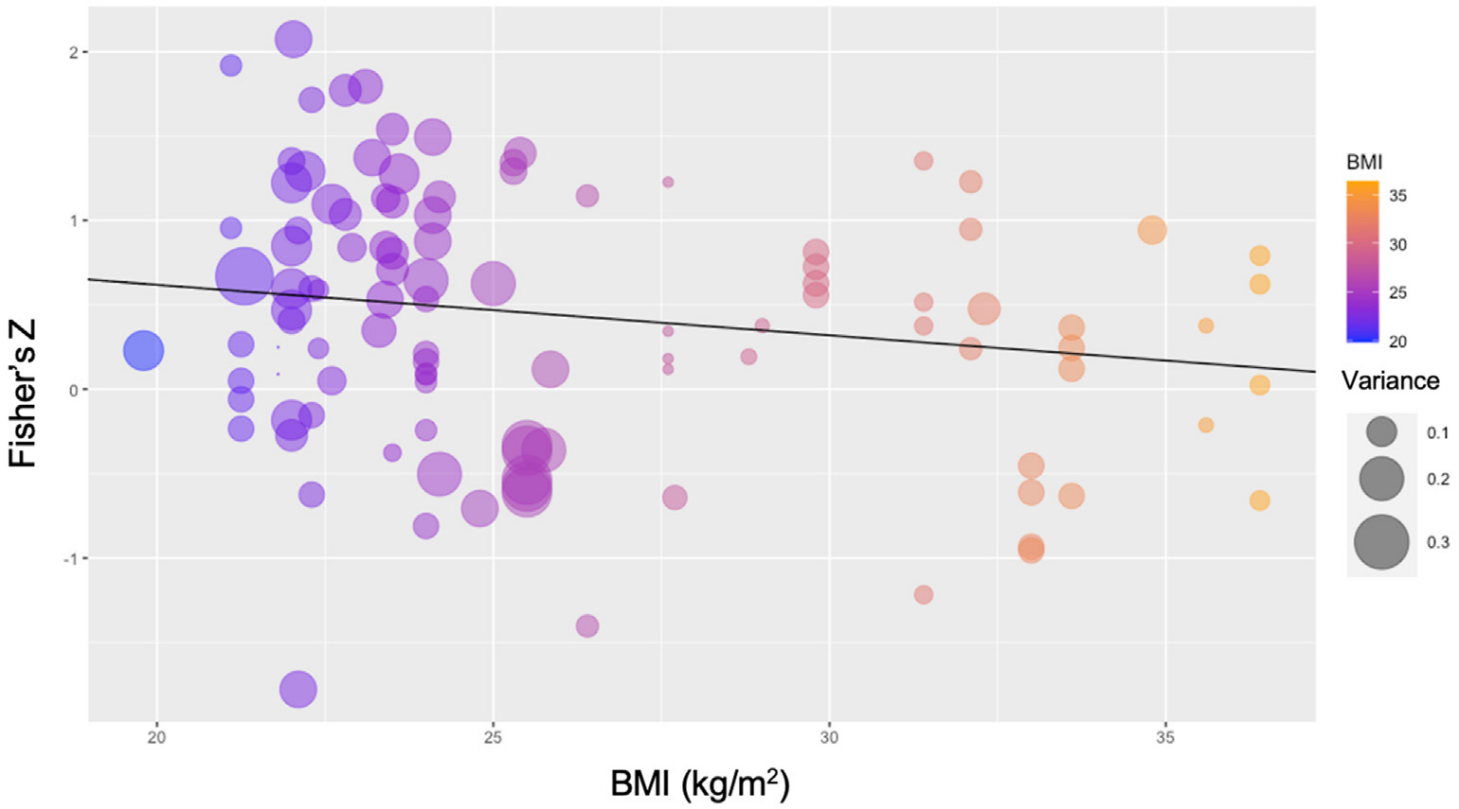
Bubble plot of the effect of BMI on the overall relationship.

Moderator data is listed in Table 2. The subgroup analyses revealed significant moderation by ghrelin form, where AG (*r* = 0.60, *P* < 0.001) and TG (*r* = 0.215, *P* = 0.01), but not DAG (*r* = 0.53, *P* = 0.695, Figure 2) moderated the overall association. Sex trended toward significance, where males (*r* = 0.65, *P* < 0.08) but not females (*r* = 0.02, *P* = 0.53) had a moderating effect on the association between ghrelin concentrations and hunger. Concerning meta-regressions, age (ß = –0.02, *P* = 0.02), BMI (ß = –0.03, *P* = 0.05, Figure 4), %BF (b =–0.03, *P* = 0.05), EI (b = 0.003, *P* = 0.04), and %CHO (ß = 0.008, *P* = 0.05) were significant, whereas %FAT and %PRO were both not significant (both, *P* > 0.05).

**TABLE 2:** Moderator analysis

Subgroup	Correlation coefficient		*P* value
Sex	Male:	*r* = 0.62 CI: –0.43, 0.83	0.08
	Female:	*r* = 0.19 CI: –0.37, 0.63	0.53
Ghrelin form	AG:	*r* = 0.60 CI: 0.41, 0.74	<0.001
	DAG:	*r* = 0.48 CI: –0.30, 0.53	0.695
	TG:	*r* = 0.24 CI: –0.37, 0.70	0.01
CHO timing	<3 h:	*r* = 0.03 CI: –0.32, 0.38	0.85
	≥3 h:	*r* = 0.51 CI: 0.31, 0.76	0.01

Meta regression	Beta coefficient		*P* value
Age		b = –0.02 CI: –0.03, –0.004	0.01
BMI		b = –0.03 CI: –0.7, 0.0006	0.05
%BF		b = –0.03 CI: –0.055, 0.0003	0.05
EI		b = 0.0003 CI: 0.0, 0.0006	0.04
%CHO		b = 0.008 CI: –0.0002, 0.02	0.05
%FAT		b =0.0035 CI: –0.006, 0.01	0.47
%PRO		b =–0.008 CI: –0.02, 0.003	0.16

AG, acylated ghrelin; DAG, de-acylated ghrelin; TG, total ghrelin; %BF, percentage of body fat; %CHO, percentage of carbohydrates; %FAT, percentage of fat; %PRO, percentage of protein.

## Discussion

### Overall and moderators

The purpose of this systematic review and meta-analysis was to investigate the relationship between endogenous ghrelin concentrations and perception of hunger in healthy adults. We report that ghrelin concentrations display a moderate, positive relationship with subjective hunger ratings. Importantly, our moderator analysis revealed that ghrelin form may influence this relationship: studies measuring AG increased the strength of the overall association, while studies measuring TG had the opposite effect, and DAG had no effect. However, this may have been impacted by the low number of trials (*n* = 12) that measured DAG. Since DAG contributes a larger percentage to plasma TG, the moderating effect we report with TG may suggest that DAG also has a weaker association with hunger ratings. Furthermore, while GHSR1a receptors, only utilized by AG, are found in the hypothalamus [8], there is no evidence of DAG receptors in the hypothalamus. Therefore, AG may be the only isoform that interacts with the appetite center in the brain. Moreover, limited evidence suggests that DAG may influence hunger by blocking AG-induced hypothalamic neuronal activity involved in appetite and food intake [71,72]. Future research should study both ghrelin isoforms individually in order to characterize their unique effects.

We report age as another significant moderator, decreasing the strength of the association between ghrelin concentrations and perception of hunger. Data suggests that hunger and food intake decrease with age, a phenomenon known as the anorexia of aging in geriatric adults [73,74]. This effect may be due to decreased gastric emptying rate and/or pyloric motility [75,76]. However, the data regarding ghrelin concentrations and age are conflicting. A meta-analysis found no differences in fasting, postprandial AG, and postprandial TG concentrations between older and younger adults [74], but the analysis included <10 studies to compare each ghrelin isoform. Findings of individual studies were equivocal, with reports of a decrease in AG [77], fasting TG [78], or comparable concentrations of each isoform [79]. Importantly, the mean age of participants in the current analysis was 31.4 ± 12.0 y; thus, there is likely not enough data in this analysis to draw conclusions on how age affects the relationship between ghrelin and perceived hunger.

We also found that sex was a moderator that trended toward significance, specifically that males impacted the relationship between ghrelin and hunger, while females did not. However, the majority of studies (*n* = 52; 47.3%) only sampled males, which suggests that our ability to assess this moderator was underpowered. Although there is a wealth of literature describing sex differences in eating behavior, such as food choices, dietary restraint [80], and EI [81], data suggest that there are no differences in the perception of appetite/hunger between males and females [80,82,83], which includes data on the hypothalamic response to hunger [84]. In contrast, prior studies have shown that there is a sex difference in plasma ghrelin concentrations. More specifically, females have been found to have higher DAG than males, a relationship that persists even when comparing individuals who are lean with those with obesity [85,86]. Furthermore, a twin study also showed that TG was higher in females; however, this difference was absent in the cohort with obesity [87].

Indeed, obesity status and body composition have been shown to impact AG concentrations and perceptions of hunger. We report that BMI and %BF were significant moderators—the average BMI of included trials was 26.0 ± 4.5. Both variables had an equal and negative effect on the overall association. The bubble plot of the BMI moderator analysis (Figure 4) illustrates that the trials with smaller variances often remained closer to the regression line than those with larger variances, suggesting that the relationship may have been stronger if there were more trials with precise measurements. Ghrelin has been suggested to modulate body weight control via a negative feedback loop [9]; individuals with obesity have been shown to experience less suppression of AG following a meal compared to non-obese individuals [88]. Moreover, a meta-analysis investigating brain activation and appetite perception in individuals with obesity found that they are more sensitive to hunger and less sensitive to satiety (feeling full) compared to individuals with normal weights [89]. However, it is important to note that the majority of included trials in our analysis did not sample individuals with elevated body weight status.

Interestingly, TG and AG have been shown to remain elevated following a meal in individuals with obesity, unlike in lean individuals, where postprandial TG and AG decrease [90,91]. This suggests a complex interplay between adiposity, ghrelin concentrations, and EI. Although we were unable to examine the relationship between ghrelin and obesity, we found that EI strengthened the overall relationship between hunger and ghrelin concentrations. This supports the findings of other studies in that exogenous AG administration has been shown to increase EI [17,18,92]. Conversely, increased EI has been shown to lead to decreased perceptions of hunger, although this may be influenced by meal macronutrient content [93,94].

Evidence suggests meal macronutrient content mediates TG, AG, and appetite levels [4,93], and we report a significant moderating effect of %CHO, where increases to %CHO strengthens the overall relationship. Considering that the average meal content in our analysis was 55.9% CHO, 25.1% FAT, and 16.9% PRO, we were likely unable to adequately assess the influence of fat and protein content on our model. Protein is suggested to have a stronger satiating effect than carbohydrates and fat [93,95], whereas the ingestion, smell, and sight of a high-carbohydrate meal have all been shown to stimulate hunger [96]. Concerning ghrelin concentrations, 1 study described a biphasic response between carbohydrate ingestion and ghrelin concentrations in adults, where AG and TG concentrations decreased during the first 3 h post-ingestion, which was then followed by an overshoot to greater than pre-ingestion levels during the following 3 h (3–6 h post-meal consumption) [4]. The average sampling time for our included trials was 3.85 ± 3.0 h, and most of the trials incorporated mixed meals. To investigate this further, we conducted an exploratory moderator analysis (Table 2) where we coded any trial that included carbohydrates in their meals with sampling times as “less than 3 h” or “more than or equal to 3 h”. The trials that sampled carbohydrates in the latter group increased the overall correlation to 0.51, whereas the trials that sampled carbohydrates for <3 h were not significant, although the correlation value was 0.03. These results not only strengthen the evidence for a biphasic carbohydrate response but also suggest that meal timing may affect the relationship between hunger and ghrelin concentrations.

### Limitations

The substantial heterogeneity and power of currently available studies denote a level of caution when translating the result of this analysis. The majority of studies had a different primary aim different than examining ghrelin concentrations and hunger and may therefore be underpowered to assess this relationship. Our sunset plot revealed that the association between ghrelin concentrations and hunger may be closer to 0.40. The majority of data points used to calculate the correlations in this analysis were measured using ImageJ, as many study authors did not respond to requests for data or no longer had access to the needed dataset. In addition, we limited our inclusion criteria to studies published in the English language, and results are restricted to healthy populations, as we also excluded major diseases. It is also important to acknowledge the volatility of sampling ghrelin. Blood samples require a protease inhibitor to ensure AG does not degrade [97]. Studies in this analysis reported a variety of blood collection methods; therefore, we cannot rule out that blood collection and processing methodology may have impacted our results. Lastly, we recognize that including multiple trials from 1 study may contribute to analytical issues such as “double counting”[98]; however, we feel our choice to apply a nested model helped reduce this effect.

In conclusion, our results suggest that ghrelin concentrations and perception of hunger exhibit a moderate, positive relationship in healthy adults. Importantly, the form of ghrelin measured (TG, DAG, or AG) impacts this correlation, along with age, BMI, %BF, EI, macronutrient content, and timing of study meals. This review highlights several gaps in the literature. Future work should be dedicated to the study of the relationship between perceptions of hunger and ghrelin concentrations as a primary aim, as most of the studies included in the current analysis were underpowered to investigate this relationship. In addition, the individual forms of ghrelin should be evaluated and should include samples that contain both sexes across the lifespan, with particular consideration to individuals with obesity. As both ghrelin and the perception of hunger are involved in regulation of body composition, meal timing, and EI, exploring the mediators of this relationship is also critical for metabolic health and weight management interventions.

### Author contributions

The authors’ responsibilities were as follows – KCA: conceptualized the project, analyzed data, performed the statistical analysis, and wrote the paper; FH and EEG: conceptualized the project, analyzed data, and wrote the paper; SK: conceptualized the project, and wrote the paper, and all authors: read and approved the final manuscript.

### Conflict of interest

The authors report no conflicts of interest.

### Funding

The authors reported no funding received for this study.

### Data availability

Data in the manuscript are available upon request.

## Appendix A Supplementary data

Multimedia component1 Multimedia component1
