# Supplementary figures and images for: Endogenous Ghrelin Levels and Perception of Hunger: A Systematic Review and Meta-Analysis
Source: Adv Nutr. 2023 Aug 2;14(5):1226–36. doi: 10.1016/j.advnut.2023.07.011 (PMC10509419; doi:10.1016/j.advnut.2023.07.011)

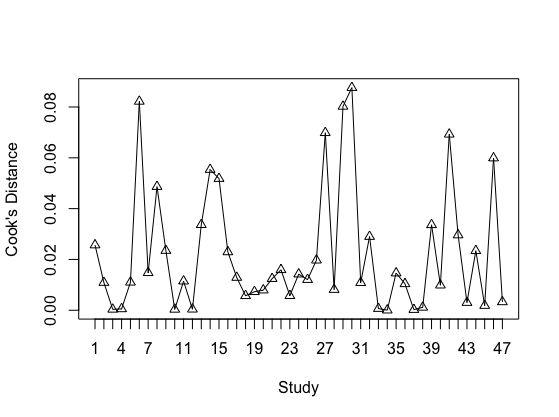

Supplement: Multimedia component1 [file mmc1.docx]
